# The Involvement of Long Non-Coding RNAs in Bone

**DOI:** 10.3390/ijms22083909

**Published:** 2021-04-10

**Authors:** Cinzia Aurilia, Simone Donati, Gaia Palmini, Francesca Miglietta, Teresa Iantomasi, Maria Luisa Brandi

**Affiliations:** 1 Department of Experimental and Clinical Biomedical Sciences, University of Florence, 50134 Florence, Italy; aurilia.cinzia@gmail.com (C.A.); simone.donati@unifi.it (S.D.); gaia.palmini@unifi.it (G.P.); francesca.miglietta@unifi.it (F.M.); teresa.iantomasi@unifi.it (T.I.); 2 Fondazione Italiana Ricerca sulle Malattie dell’Osso (FIRMO Onlus), 50141 Florence, Italy

**Keywords:** lncRNAs, osteoblastogenesis, osteoclastogenesis, bone-related disorders, precision medicine

## Abstract

A harmonious balance between osteoblast and osteoclast activity guarantees optimal bone formation and resorption, pathological conditions affecting the bone may arise. In recent years, emerging evidence has shown that epigenetic mechanisms play an important role during osteoblastogenesis and osteoclastogenesis processes, including long non-coding RNAs (lncRNAs). These molecules are a class of ncRNAs with lengths exceeding 200 nucleotides not translated into protein, that have attracted the attention of the scientific community as potential biomarkers to use for the future development of novel diagnostic and therapeutic approaches for several pathologies, including bone diseases. This review aims to provide an overview of the lncRNAs and their possible molecular mechanisms in the osteoblastogenesis and osteoclastogenesis processes. The deregulation of their expression profiles in common diseases associated with an altered bone turnover is also described. In perspective, lncRNAs could be considered potential innovative molecular biomarkers to help with earlier diagnosis of bone metabolism-related disorders and for the development of new therapeutic strategies.

## 1. Introduction

The correct function of the skeleton is guaranteed by a continuous optimal remodeling of bone tissue throughout life, characterized by a balance between the mechanisms of osteoclastogenesis and osteoblastogenesis [1]. The bone formation process involves the production, maturation, and mineralization of the bone matrix. Osteoblasts are responsible for the formation of bone matrix constituents. They originate from mesenchymal stem cells, which, to differentiate into osteoblasts, require the expression of the *runt-related transcription factor 2* (*RUNX2*) and *osterix* (*OSX*) genes [2]. The activity of osteoblasts is regulated in an autocrine and paracrine manner by different hormones, such as bone morphogenetic proteins (BMP) [3], basic fibroblast growth factor (bFGF) [4], and in an endocrine manner by hormones, such as prolactin [5] and parathyroid hormone (PTH). In contrast, osteoclasts are responsible for bone resorption. They originate from monocytes that express on the surface the activator of NF-kappa B receptor (RANK), which interacts with RANK ligand secreted by osteoblasts leading to differentiation in osteoclasts [6]. These cells are then able to adhere to the bone surface and begin resorption, by secreting proteolytic enzymes, such as cathepsin K [7]. When the balance between destruction and deposition of a new mineralized bone matrix is altered, several bone diseases may arise. In particular, excessive bone resorption is the main cause of several diseases, such as osteoporosis (OP) and bone fragility, caused by the loss of bone mass. OP occurs predominantly in postmenopausal women, and it is estimated that around nine million people worldwide suffer osteoporotic fractures each year, making this disease a major problem from the point of view of morbidity, mortality, and healthcare costs. Despite the existence of diagnostic tools aimed at assessing the risk of fracture, these have limitations that restrain their use in clinical practice, suggesting that there is a need to find new biomarkers for rapid diagnosis, as well as new guidelines for OP treatment [8]. At the same time, reduced bone resorption is the basis of the onset of another bone disease, osteopetrosis, a genetic disease caused by osteoclast activity failure, causing the closure of bone cavities and foramina with subsequent hematological failure and nerve compression, respectively [8]. In addition, mutations in bone cells are responsible for the origin of bone tumors, such as osteosarcoma (OS) and Ewing’s Sarcoma (ES). These are the two most common cancers of the primary bone cancers, which occur mainly in children, adolescents, and young adults. Both tumors present a high degree of malignancy and are clinically characterized, in most circumstances, by systemic metastasis and a significant rate of relapse following an initial response to the multimodal treatments [9,10]. The current absence of a specific molecular signature with prognostic significance in OS and ES treatments stimulates the scientific community to identify novel biomarkers for the development of more effective therapeutic and diagnostic strategies against these tumors. In the light of the latest studies showing that osteoblastogenesis and osteoclastogenesis may not be only regulated by genetic factors, but also by epigenetic factors, an alteration in the latter may be at the base of diseases associated with changes in bone turnover. [11].

Epigenetics refers to the study of hereditable, reversible changes in gene expression that do not affect DNA sequences, and includes the investigation of histone modifications, DNA methylation, and noncoding RNAs (ncRNAs)-mediated transcriptional regulation [11].

DNA methylation consists of a specific addition of a methyl group (-CH3) to the C5 position of the cytosine ring of DNA (5mC), mainly within CpG islands, led by DNA methyltransferases (DNMTs), and generally linked to the silencing of gene expression [12].

Histone modification is an epigenetic mechanism that occurs by post-translational modification of histone proteins, including acetylation, methylation, ubiquitination, and phosphorylation, resulting in an altered degree of chromatin accessibility [12].

Historically, ncRNAs were considered as “junk RNAs” with no biological function. However, with the recent advances in more efficient, cost-effective, and sensitive sequencing techniques, it has been observed that only 1–2% of the human genome consists of protein-coding genes, while the largest part is represented by non-coding genome with a huge role in several molecular mechanisms. ncRNAs can be categorized according to their RNA molecule length: short ncRNAs, smaller than 200 nucleotides (i.e., Piwi-interacting RNAs (piRNAs), small interfering RNAs (siRNAs), small nuclear RNAs (snRNAs), small nucleolar RNAs (snoRNAs), and microRNAs (miRNAs)) and nc transcripts >200 nucleotides in length, referred to as long non-coding RNAs (lncRNAs) [13].

In recent years, increasing evidence has pointed to miRNAs, short non-coding RNAs about 18–25 nucleotides long with a negative role of gene expression control, and lncRNAs, non-protein-coding transcripts of approximately 200 or more nucleotides in length, as potential novel diagnostic and therapeutic biomarkers for a wide range of diseases [14].

In 1980, researchers focused their efforts on studying known protein-coding genes by using molecular cloning systems and analysis of spatial-temporal patterns of gene expression. During those years, the first lncRNA was identified, namely H19, initially classified as mRNA, whose expression was strictly regulated by genomic imprinting together with Insulin-like growth factor 2 (Igf2) as a part of a dosage compensation mechanism in transgenic mice [15]. Its function was not fully elucidated until the discovery in the early 1990s of X-inactive-specific transcript (XIST), a lncRNA considered to be indispensable for the X chromosome inactivation (XCI) throughout early embryonic development in mammals [15].

Since the conclusion of the Human Genome Project (HGP), a 13-year international research effort to determine the complete mapping of the human DNA sequence, multiple genes, including lncRNAs, have been identified and characterized in several organisms, thus revealing their new biological and functional roles in various biological processes [16,17,18].

In the same way as protein-coding mRNAs, many lncRNAs are transcripted by RNA polymerase II in the nucleus and are processed by 5′ capping, 3′ poly-A tail addition, and RNA splicing [19].

Compared with short ncRNAs and protein-coding genes, this family of long transcripts has been proven to be less conserved across different species [20,21]. Nevertheless, lncRNAs exhibit conserved promoter regions and splice sites, thus indicating that they could be functionally related genes, independent of their different sequence conservation [20,21].

So far, over seventeen thousand human lncRNAs have been listed in the Human GENCODE database [GENCODE. Available online: https://www.gencodegenes.org/ (accessed on 10 December 2020); Release Version 36: May 2020] [22].

These long ncRNA molecules are classified into two major subgroups, intergenic and intragenic lncRNAs, in relation to their genomic positions [23]. The intergenic, also termed lincRNAs, are lncRNAs placed within intergenic regions that do not overlap with protein-coding regions. On the other hand, intragenic lncRNAs overlap with protein-coding regions and can be divided into four additional categories, depending on their location in the relative protein-coding gene [24]:

- lcRNAs that overlap with one or more exonic or intronic or both regions and are transcripted from the same strand of a coding gene (Sense lncRNAs);
- lncRNAs that overlap in a similar way of sense lncRNAs but are transcripted on the opposite strand (Antisense lncRNAs);
- lncRNAs that originate from the opposite direction although sharing the same promoter of the protein-coding gene (Bidirectional lncRNAs);
- lncRNAs derive totally from introns within coding genes (Intronic lncRNAs).

Furthermore, it has been demonstrated that intronic and sense lncRNAs could form circular lncRNAs (circRNAs) through the formation of a covalence bound between 3′ and 5′ ends of the RNA molecule during noncanonical splicing events, leading to the generation of a circular closed continuous single-stranded RNA [25].

It has been established that these molecules are located within the cytoplasm, or remain, in most circumstances, in the nucleus, forming in both cases thermodynamically stable structures [14].

**Figure 1:**
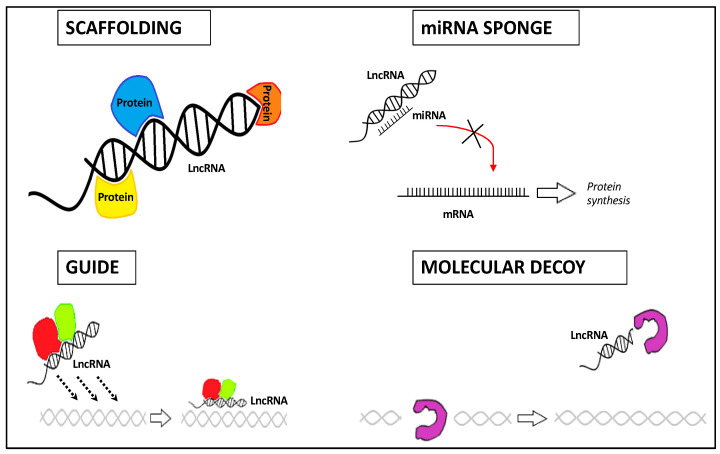
Four main regulatory mechanisms of lncRNAs by which these molecules affect gene expression by exerting scaffolding, sponge, guide, and decoy function.

Based on their compartment localization and structure, lncRNAs have specific functions as regulators of transcription, post-transcription, and/or translation, serving as scaffold, guide, decoy, and sponge ncRNA molecules [26,27] (Figure 1):

- Their structural plasticity offers interaction sites with a high affinity for proteins, DNAs, or RNAs, thus forming ribonucleoprotein complexes (lncRNA scaffold);
- Depending on the biological context, they can conduct ribonucleoprotein complexes towards a certain genomic location (lncRNA guide);
- lncRNAs can interact with protein complexes, thereby blocking or enhancing protein activities (lncRNA decoy);
- lncRNAs can act as competing endogenous RNAs (ceRNAs) by binding directly and sequestering the miRNA, thus decreasing its regulatory effect on mRNA targets (lncRNA sponge).

Increasing evidence has indicated that lncRNAs, as critical regulators of gene expression, could be involved in osteoblast and osteoclast proliferation, differentiation, apoptosis, and activity [28]. As a dysregulation of their expression patterns has been demonstrated to be involved in the development of a wide range of diseases (i.e., aging, cancer, metabolism diseases, and OP) [29,30,31,32,33], recent studies have aimed to elucidate their roles during osteoblastogenesis and osteoclastogenesis processes. Understanding the role of LncRNAs could pave the way for the development of novel lncRNA-based strategies to improve diagnosis and prognosis, and for new treatment of diseases associated with bone turnover.

In this review, we aim to provide a general overview of the current evidence of the involvement of lncRNAs in osteoblastogenesis and osteoclastogenesis, as well as their role in metabolic bone diseases related to the disequilibrium between these two processes. A systematic review was performed to select articles published over the last two years (2019–2020) regarding lncRNAs involved in osteoblastogenesis and osteoclastogenesis using combinations of keywords, such as “lncRNAs”, “osteoblastogenesis”, “osteoblasts”, “osteoclastogenesis”, and “osteoclasts”. In the second section, we review knowledge on the most promising lncRNAs in three common bone metabolic disorders, such as OS, ES, and OP, obtained within the last five years (2015–2020).

## 2. LncRNAs Regulating Osteoblastogenesis

### 2.1. LncRNAs as Positive Regulators

After reports of the role of prostate cancer-associated ncRNA transcript-1 (PCAT1) in osteogenic differentiation of adipose-derived stem cells, Jia et al. [34] further investigated its possible mechanism of action in osteogenic differentiation of periodontal ligament stem cells (PDLSCs). By profiling lncRNA expression analysis by using a macro array platform, the authors found that lncRNA PCAT1 was increased in PDLSC osteogenic-induced, compared to non-induced, cell groups. It was also found that the overexpression of lncRNA PCAT1 increased Alkaline Phosphatase (ALP) activity and hydroxyapatite crystal formation. The effect of its overexpression was further evaluated in Nonobese diabetic/severe combined immunodeficiency (NOD/SCID) mice, thus showing an increased osteoid formation compared to the negative control, while the knockdown of lncRNA PCAT1 decreased osteoid formation. The authors then studied the interaction between lncRNA PCAT1 and mir-106a-5p by luciferase assay. They found that lncRNA PCAT1 could sponge mir-106a-5p by leading to upregulation of its target gene *BMP2*, resulting in positive regulation of osteogenic differentiation. Among the 137 predicted target genes of mir-106a-5p, revealed by bioinformatics analysis, only the *E2F5* gene was confirmed by luciferase reporter assay. Therefore, they identified that this transcription factor could interact with lncRNA PCAT1 promoter, forming a feed-forward regulatory network targeting BMP2, suggesting that this novel mechanism could represent a promising target to regulate the osteogenic differentiation of PDLSCs.

In 2019, Yuan et al. [35] identified a novel lncRNA, the peroxisome proliferator-activated receptor γ coactivator-1β-OT1 (PGC1β-OT1). Authors found altered expression levels of this lncRNA during adipogenic and osteogenic differentiation in progenitor cells. In fact, by using quantitative RT-PCR (qPCR), they have seen that lncRNA PGC1β-OT1 expression drastically increased at an earlier stage and decreased at a later stage of adipogenesis in bone marrow-derived stromal ST2 cells. To study the functional role of lncRNA PGC1β-OT1 during adipogenesis and osteogenesis, ST2 cells transfected with PGC1β-OT1 overexpression construct were used, finding that overexpression of this lncRNA could inhibit adipogenic and increase osteogenic differentiation. Opposite results were seen with lncRNA PGC1β-OT1 silencing. Furthermore, the authors found that lncRNA PGC1β-OT1 may act as a sponge of miR-148a-3p, through their physical association, by regulating the expression of its target gene *lysine-specific demethylase 6 b* (*KDM6B*). Interestingly, they found that lncRNA PGC1β-OT1-miR-148a-3p association led to adipocyte formation decrease, while supplementation of miR-148a-3p in progenitor cells arrested PGC1β-OT1 inhibitory effect on adipogenesis. Finally, the authors found that the overexpression of KDM6B promoted osteogenic differentiation. In conclusion, this study evidenced that lncRNA PGC1β-OT1 could regulate adipogenesis and osteogenesis, exerting a direct negative effect on miR-148a-3p and enhancing KDM6B action.

Recently, the novel lncRNA OGRU was identified by Wang et al. [36]. They found that this lncRNA was downregulated in osteoblastic cell line MC3T3-E1 cultured in unloading condition. Its expression levels were also observed to be decreased in the tibia of hindlimb unloaded (HLU) mice. On the contrary, lncRNA OGRU was upregulated during osteoblast differentiation in MC3T3-E1 cells cultured in an osteogenic medium. To investigate the possible involvement of lncRNA OGRU in osteogenesis, the authors used MC3T3-E1 cells transfected with pcDNA3.1(+)-OGRU vector. They discovered that lncRNA OGRU overexpression could attenuate the reduction of osteogenic markers genes in pcDNA3.1(+)-OGRU cells cultured in clinorotation unloading condition. Furthermore, the function of this lncRNA in osteogenesis was also investigated in vivo, by using HLU mice treated with pcDNA3.1(+)-OGRU delivered by (DSS)6-liposome to the bone formation surface, showing that this treatment led to a rescue of unloading-induced bone loss. Finally, they demonstrated that lncRNA OGRU acts as a ceRNA of miR-320-3p to promote Hoxa 10 protein expression and consequently bone formation.

The purpose of a study by Wang et al. [37] was to elucidate the effect of lncRNA AK023948 (AK0) in postmenopausal OP rats. First, the authors saw that the PI3K/AKT signaling pathway was downregulated in OP rats compared to the control group, indicating that this pathway has a central role in OP occurrence and development. They then evaluated the lncRNA AK0 action on AKT activity, discovering that lncRNA AK0 overexpression leads to an increase of AKT phosphorylation levels, while lncRNA AK0 expression interference reduces AKT phosphorylation levels. Therefore, these data showed that lncRNA AK0 could play an important role in postmenopausal OP by regulating the PI3K/AKT pathway.

Given the importance of finding new prevention and therapeutic strategies for the treatment of degenerative bone disease, Cao et al. [38] identified the novel lncRNA LINC02349 and investigated its potential role in osteoblast differentiation processes. They observed a rapid and increased mineralization process in human umbilical cord-derived stem cells overexpressing lncRNA LINC02349, compared to cells with lncRNA knockdown. Based on the hypothesis that lncRNA LINC02349 could act as a ceRNA for a specific miRNA, they screened 39 miRNAs, among which miR-25-3p and miR-33b-5p were the most promising candidates. Further studies showed that lncRNA LINC02349 was able to directly sponge these two miRNAs and positively regulate their downstream targets, SMAD 5 and Wnt 10b, two key factors of the osteogenic differentiation process. Finally, the authors observed that transcription factor STAT3 promotes lncRNA LINC02349 expression through its association with ligand-bound glucocorticoid receptor, which was recruited to the promoter region of this lncRNA.

Emerging evidence of lncRNA KCNQ10T1 involvement in osteogenic processes have encouraged Chen and his group [39] to explore the role of this lncRNA during osteoblast migration, proliferation and survival, and, therefore, fracture healing. A study about the activity of lncRNA KCNQ10T1 in MC3T3-E1 cells showed that its knockdown leads to cell proliferation suppression with respect to cells with a normal expression of lncRNA KCNQ10T1. In addition, a decrease in cell migration and higher rates of apoptosis were observed in KCNQ10T1-silenced cells. By using the mouse femur fracture model, authors have seen that lncRNA KCNQ10T1 levels increased during fracture healing, hypothesizing that it could be involved in this process. Based on previous studies about the interactions between lncRNA and miRNA, they investigated the potential relationship between lncRNA KCNQ10T1 and miR-701-3p, by using RNA immunoprecipitation and pull-down assay. The results showed that lncRNA KCNQ10T1 acted as a sponge of miR-701-3p, by regulating its target gene *FGFR3*, which promotes bone formation. In conclusion, the authors demonstrated that lncRNA KCNQ10T1 promotes osteoblast proliferation, migration, and survival through miR-701-3p/FGFR3 axis regulation.

The role of lncRNA KCNQ10T1 was also studied by Wang et al. [40]. They investigated whether KCNQ1OT1 may affect osteogenic differentiation by sponging miR-214. The qPCR analysis reveals that expression levels of KCNQ1OT1 and osteoblast-related genes (*RUNX2*, *BMP2*, *osteopontin* (*OPN)*, and *osteocalcin* (*OCN)*) are upregulated, while those of miR-214 are markedly downregulated during osteogenesis. KCNQ1OT1 silencing in human bone marrow mesenchymal stem cells (hBMSCs) decreases ALP activity and matrix mineralization, as well as osteogenesis-related marker genes and protein levels, while miR-214 expression is notably upregulated, resulting in an inhibition of osteoblast differentiation. By using in silico tools, a dual luciferase reporter assay, and in vitro experiments, the authors observed that BMP2 expression and its downstream Smad signaling could be under the control of KCNQ1OT1, which could directly interact with miR-214 in hBMSCs, demonstrating the role of the KCNQ1OT1/miR-214/BMP2 axis in regulating the osteoblast differentiation process.

The aim of a study by Li et al. [41] was to investigate the role of lncRNA LOC100506178 in hBMSCs osteogenic differentiation. They found that this lncRNA is upregulated in hBMSCs during osteogenic differentiation. Further studies demonstrated that lncRNA LOC100506178 acted as a sponge of miR-214-5p and positively regulated its target gene *BMP2*, which is crucial for bone formation. Therefore, these data showed the involvement of this new molecular axis consisting of lncRNA LOC100506178/miR-214-5p/BMP2 in the hBMSCs osteoblast differentiation process.

Jia et al. [42] studied the role of lncRNA LINC00707 in hBMSCs during the osteogenic differentiation process. They reported that its expression levels increase during this process, suggesting a positive role of LINC00707 on bone formation. In addition to this, subsequent studies revealed that lncRNA LINC00707 could act as a ceRNA for miR-370-3p, which has been identified as a negative regulator of osteogenic differentiation. Moreover, Jia et al. discovered that lncRNA LINC00707 overexpression leads to Wnt2B increase, a miR-370-3p gene target, and a key positive regulator of osteoblast differentiation. Therefore, these results exhibited the existence of a new lncRNA-miRNA regulatory network, providing a promising target to regulate hBMSC osteogenic differentiation.

The aim of a study by Liu et al. [43] was to elucidate the lncRNA Taurine Up-regulated Gene 1 (TUG1) function in osteogenesis. First, the authors discovered that lncRNA TUG1 and Wnt/β-catenin pathway markers have the same increasing trend during osteoblast differentiation, indicating that lncRNA TUG1 could be involved in this mechanism. Further studies showed that lncRNA TUG1 silencing, by using short hairpin TUG1 (sh-TUG1), leads to a lack of osteoblast proliferation and differentiation and Wnt/β-catenin pathway-related protein reductions. Therefore, they demonstrated that lncRNA TUG1 inhibition could inhibit the osteogenic process through Wnt/β-catenin signaling pathway suppression, suggesting that this lncRNA could act as a positive regulator of bone formation.

Hao et al. [44] also investigated its role in the osteoblast precursor cells hFOB1.19 proliferation and differentiation. The effect of lncRNA TUG1 on cell proliferation was explored by using hFOB1.19 cells transfected with lncRNA TUG1 or si-TUG1. They observed that cells overexpressing TUG1 show an increment of cell viability with respect to untreated cells, while the si-TUG1 group revealed a cell viability suppression. TUG1 overexpression in hFOB1.19 also increases ALP activity and osteoblastic differentiation marker genes, inducing osteoblast differentiation. Furthermore, hFOB1.19 cells cultured with osteogenic medium present increased expression levels both of lncRNA TUG1 and cannabinoid receptor 2 (CNR2), while a reduction in miR-545-3p expression levels was observed. Moreover, osteogenic-related protein levels and ALP activity increase in hFOB1.19-OM cells, compared to control cells. Subsequently, the bioinformatic analysis predicted that lncRNA TUG1 could also be capable of binding miR-545-3p, and this data has been confirmed by luciferase and RNA immunoprecipitation assays. Further studies have reported that *CNR2* is one of the target genes of miR545-3p. In fact, its depletion in cells treated with miR-545-3p reduces osteogenic-related gene expression, whereas the introduction of anti-miR-545-3p recovers this effect. In conclusion, this study revealed that lncRNA TUG1 could facilitate the proliferation of hFOB1.19 cells and promote their differentiation by sponging miR-545-3p and upregulating its gene target *CNR2*.

Wang et al. [45] investigated the role of lncRNA growth arrest-specific transcript 5 (GAS5) during osteogenic differentiation, discovering that lncRNA GAS5 expression levels are lower in the BMSCs derived from ovariectomized (OVX) mice compared to that isolated from the control group. Further studies revealed that lncRNA GAS5 overexpression is capable of increasing mRNA and protein levels of osteogenic marker genes (i.e., RUNX2, Col1a1, and OCN), and increasing ALP activity and hydroxyapatite crystal formation. On the contrary, lncRNA GAS5 downregulation leads to opposite results, indicating that this lncRNA may be able to promote BMSC osteogenic differentiation. Later, the authors predicted the possible bind between lncRNA GAS5 and miR-135a-3p by using bioinformatic programs and confirmed it by using a dual luciferase reporter assay. It was observed that lncRNA GAS5 promotes osteoblast differentiation through miR-135a-3p expression reduction and by regulating its gene target *forkhead box O1* (*FOXO1)* expression level. Therefore, the authors demonstrated that lncRNA GAS5 acts as a ceRNA for miR-135a-3p, thereby upregulating FOXO1 expression and stimulating osteoblast differentiation.

Recently, Yang et al. [46] tried to understand the further role of lncRNA GAS5 in osteogenic differentiation of human PDLSC (hPDLSCs). First, lncRNA GAS5 expression during osteogenic differentiation was evaluated by using qPCR, finding that hPDLSCs cultured in the osteogenic medium show an upregulation of lncRNA GAS5 expression and growth differentiation factor 5 (GDF5), a crucial regulator of the osteogenesis process in the extracellular matrix. Moreover, the levels of osteogenic markers mRNA are also increased in hPDLSCs-OM with respect to control cells. They also reported that investigating lncRNA GAS5 mechanism of action, there is a relationship between this lncRNA and GDF5. In fact, western blotting analysis revealed that GDF5 levels are decreased and increased in cells overexpressing or downregulating lncRNA GAS5, respectively. The authors then investigated the possible regulation of MAPK signaling pathway proteins by lncRNA GAS5 and GDF5, finding that p38 and JNK phosphorylation decreases in cells with GDF5 downregulation and increases in cells with GAS5 overexpression. All these results show the ability of lncRNA GAS5 to promote osteogenic differentiation of hPDLSCs by regulating GDF5 and participating in the MAPK signaling pathway.

Considering the crucial role of RUNX2 on osteogenic differentiation, Yi et al. [47] tried to understand the effect of lncRNA metastasis-associated lung adenocarcinoma transcript 1 (MALAT1) on RUNX2 expression and, consequently, on osteogenic differentiation. First, the authors evaluated lncRNA MALAT1 and RUNX2 expression levels in human adipose-derived mesenchymal stem cells (hADMSCs) cultured in osteogenic differentiation media, finding that both factors increase, whereas miR-30 expression decreases. Subsequent studies, using hADMSCs transfected with siRNA against lncRNA MALAT1 and a miR-30 overexpression vector, reported that lncRNA MALAT1 knockdown and miR-30 overexpression suppress osteogenic differentiation through osteoblastic marker gene inhibition, such as RUNX2, OCN, OSX, and osteopontin OPN. It was then established, using bioinformatic analysis and luciferase assay, that miR-30 is capable of binding both RUNX2 3′-UTR and MALAT1. In conclusion, the authors demonstrated that lncRNA MALAT1 could act as a sponge for miR-30 to promote RUNX2 expression and osteogenic differentiation.

Occlusal trauma, a pathological injury of periodontal tissue, stimulated the nuclear factor kappa-light-chain-enhancer of activated B cell (NF-kB) signaling, thus inhibiting the osteogenesis process. In this light, Lu et al. [48] investigated the role and the underlying molecular mechanisms of differentiation antagonizing non-protein coding RNA (DANCR) in the inhibition of this process. The results showed that the traumatic compressive stress-induced inhibition of osteogenic differentiation is promoted by the activation of the NF-kB pathway through the downregulation of DANCR in MC3T3-E1 cells, while the induced overexpression of such lncRNA increases the expression of *ALP* and *RUNX2* genes, thus alleviating the inhibitory effect induced by traumatic stress.

Xiong et al. [49] studied whether the lncRNA Rhno1/miR-6979-5p/BMP2 axis could be involved in the regulation of osteoblast differentiation. They observed that the expression levels of miRNA-6979-5p are deregulated between the calluses obtained from the femoral fracture mouse model and mouse models with normal bone. In addition, its overexpression is negatively correlated to osteoblast differentiation. Through bioinformatic analysis and luciferase reporter assays, they identified the *BMP2* gene, previously described in many studies as a positive regulator gene of osteogenic differentiation, to be a target of miR-6979-5p. In vitro and in vivo experiments showed that lncRNA Rhno1 can function as a ceRNA of miR-6979-5p, resulting in an increased *BMP2* expression both in vitro and in vivo, suggesting that this lncRNA may play a positive role in the regulation of osteogenic differentiation. Finally, they evaluated the effects of Rhno1 injection on fracture healing of a fracture murine model. The results obtained showed that Rhno1 downregulates miR-6979-5p and increases BMP2 expression levels, thus promoting fracture healing in vivo.

Colorectal neoplasia differentially expressed (Crnde) has previously been reported to be a cancer-related lncRNA and to be involved in the proliferation and differentiation of osteoblasts. In relation to this, Mulati et al. [50] studied whether Crnde preserves this regulatory function in bone remodeling in vivo. In vitro experiments carried out on MC3T3-E1 and primary calvarial osteoblasts, cell lines revealed that this lncRNA is expressed in osteoblastic cell lineage and its expression is upregulated by PTH. To verify the in vivo role of Crnde, Crnde knockout mice were generated by using Clustered Regularly Interspaced Short Palindromic Repeats/Cas9 nuclease (CRISPR/Cas9) system. The authors showed that its ablation inhibits osteoblast proliferation and differentiation, resulting in low bone mass phenotype in Crnde −/− mice, while overexpression of Crnde partially restores their differentiation and promotes their proliferation by activating the Wnt/β-catenin signaling pathway. Collectively, results from this study suggested Crnde as a novel bone metabolism regulator.

In a study by Sun et al. [51], expression profiles between undifferentiated human- and mouse-derived BMSCs and osteogenically differentiated BMSCs, performed by using high-throughput RNA sequencing (RNA-seq), revealed 1436 differentially expressed lncRNAs. Among these, osteoblast-enriched lncRNA (lnc-ob1) was selected for further analysis, based on the results of tissue expression pattern analysis in mice, because it was remarkably higher in bone with respect to other tissues. In fact, its expression levels are markedly higher in osteoblasts and upregulate during the osteoblastogenesis process. Conversely, it was found significantly downregulated in osteoblasts derived from OVX mice compared to the control group. Increased bone formation and bone mass, as well as elevated bone mineralization, were observed in knock-in mice overexpressing lnc-ob1and in human osteoblastic cell lines, respectively. Furthermore, pharmacological osteoblast-specific administration of lnc-ob1 preserves the progressive loss of bone in an OVX mouse model. In vitro analysis performed in mouse and human osteoblasts showed that lnc-ob1 promotes osteoblast differentiation and osteogenesis by increasing OSX expression through the inhibition of OSX promoter methylation guided by H3K27me3. This study, therefore, indicates lnc-ob1 as a potential molecular target for the treatment of OP.

Given that *RUNX2* has been reported to be a downstream gene of the PTH signaling responses during osteogenesis, the aim of a study by Arumugam et al. [52] was to investigate the in vitro effects of PTH on the regulation of osteoblast differentiation mediated by RUNX2 and ncRNAs. Rat PTH treatment induces higher *RUNX2* expression levels than in the respective controls at 24 h, 7 days, and 14 days. The bioinformatic analysis found 17 lncRNAs in the chromosomal region where the human *RUNX2* gene originates. Among these, the expression profile of lnc-SUPT3H-1:16 shows a similar expression pattern to *RUNX2* mRNA up to 7 days of PTH-treated hBMSCs. Using miRNA target prediction tools, 4 miRNAs (miR-6797-5p, miR-4516, miR-1249-5p, and miR-6893-5p) were indicated as putative targets of both *RUNX2* and lnc-SUPT3H-1:16. PTH treatment increases the expression levels of these miRNAs and, especially, of miR-6797-5p. Based on the results derived from MG63 cells transfected with miR-6797-5p mimic and negative control (NC), the authors observed that miR-6797-5p overexpression downregulates the RUNX2 protein expression and consequently their differentiation into osteoblasts, indicating a role for lnc-SUPT3H-1:16 as a miRNA sponge lncRNA confirmed by luciferase gene reporter assay. Taken together, these findings reveal that PTH increases the expression levels of lnc-SUPT3H-1:16, miR-6797-5p, and RUNX2. In addition, the latter is protected by the sponge function of lnc-SUPT3H-1:16 on miR-6797-5p, thus promoting osteoblast differentiation.

Wang et al. [53] investigated whether osteoblast differentiation-related lncRNA under simulated microgravity (ODSM) could promote bone formation following mechanical unloading stimuli. The authors found that ODSM could promote mineral deposition and inhibit apoptosis in the osteoblastic cell line MC3T3-E1. The relationship between ODSM and miR-139-3p, which have previously been found to interact with one another, and ELK1, a transcription factor closely related to osteogenesis, was studied in vitro. Based on their results, Wang et al. found that ODSM regulates ELK1 expression by interacting with miR-139-3p. Subsequently, they evaluated whether this lncRNA could affect osteoblast apoptosis and differentiation, and the related mechanisms, in MC3T3-E1 transfected with ODSM mimic and NC and cultured in a microgravity unloading environment. Apoptosis and osteogenesis are suppressed and increased by ODSM through interaction with miR-139-3p, respectively. In vivo analysis showed that ODSM overexpression obtained by (AspSerSer)_6_-liposome delivery system significantly increases bone formation by contrasting the decline in osteoblast activity and apoptosis in hindlimb-unloaded mouse models.

Given that LncRNA small nucleolar RNA host gene 7 (SNHG7) has been associated with many types of cancers, Chen et al. [54] investigated its expression and its potential role during the osteogenesis process in femoral neck fracture tissues. The obtained results showed that SHNG7 expression levels are remarkably downregulated in fractured tissues compared to those in normal tissues. Knockdown of SNHG7 in MC3T3-E1 cells results in reduced osteoblast activity, migration, and proliferation, as well as a promotion of programmed cell death. Using bioinformatic tools, the authors identified miR-9 as a predicted miRNA target for SNHG7; in addition, this miRNA is predicted to bind the 3′UTR of *TGFBR2* mRNA. Luciferase activity reporter assays and transfection of MC3T3-E1 with SNHG7, miR-9, or miR-9 + SNHG7 confirm both findings. Furthermore, SNHG7 silencing suppresses the expression levels of TGFBR2, p-smad2, and p-smad3, thus inhibiting the TGF-*β* signaling pathway. All these data assume that SNHG7 may play a key role in femoral neck fracture.

The aim of a study by Del Real et al. [55] was to analyze the lncRNA expression patterns in hBMSCs obtained from 9 osteoporotic patients with hip fractures (FRX) compared to that in 8 osteoarthritides (OA)-derived BMSCs, used as controls. Two independent RNA sequences were carried out on hBMSCs derived from 9 FRX and 8 OA, and 10 FRX and 10 OA, respectively, revealing an overlap for 52 differentially expressed genes, 20 of which were lncRNAs. As revealed by Spearman correlation analysis, these lncRNAs, identified to be differentially expressed, are respectively correlated with protein-coding and bone-related protein-coding genes. Finally, of the 163 differentially expressed genes (99 lncRNAs) between pre- and post-differentiation hBMSCs identified from the RNA sequencing, only two lncRNAs (LINC00341 and PACERR) were validated and confirmed by using qPCR and, in particular, LINC00341 overexpression induced upregulation of osteoblast-specific marker genes.

Chen et al. [56] studied the role of lncRNA MCF2L-AS1 in BMSC osteogenic differentiation and the underlying molecular mechanisms. Microarray analysis was performed to detect lnRNA expression during the hBMSC osteoinduction, identifying nine upregulated lncRNAs and fifteen downregulated lncRNAs, according to the absolute fold change ≥ 1.5 and *p*-value ≤ 0.05. Of the 24 differentially expressed lncRNAs, only MCF2L-AS1 was selected and verified to be upregulated during osteogenic induction of hBMSCs, compared with controls. Using bioinformatic analysis and luciferase activity assay, miR-33a has been demonstrated to be a direct target of MCF2L-AS1. Moreover, analysis of hBMSCs transfected with miR-33a mimic or silenced for MCF2L-AS1 reveals that this lncRNA stimulates osteogenic differentiation in hBMSCs by upregulating RUNX2 through suppressing the aforementioned miRNA, suggesting the lncRNA MCF2L-AS1 as an enhancer of osteogenic differentiation.

Finally, two studies evaluated whether lncRNA H19 played an important role in the osteogenesis of hBMSCs by regulating miRNA expression.

After establishing an in vitro rat-derived osteogenic differentiation model, Li et al. [57] compared H19 expression between cells cultured in osteogenic condition compared with control, revealing its increased expression in differentiated cells with respect to cells without treatment. Additional experiments showed that H19 overexpression by H19 mimic treatment induces a relevant increase of ALP activity and calcium nodule formation, as well as an increase of osteogenic-related protein levels (i.e., ALP, OCN, RUNX2, and OSX), as opposed to cells transfected with shRNA. Luciferase reporter assay verified the binding of H19 and miR-149, revealed from a previous analysis by in silico prediction tools. The study also reported that the *SDF-1* expression levels, a gene identified by bioinformatics predictions, are inversely correlated with those of miR-149 in hBMSCs transfected with miR-149 mimic. Finally, Li et al. evaluated the relationship between H19 and osteogenic differentiation on cells treated with shRNA-NC, shRNA-SDF-1, H19-NC, H19, H19 + shRNA-NC, and H19 + shRNA-SDF-1. The results obtained from this study indicate that H19 upregulation enhances osteoblast differentiation by increasing SDF-1 expression by concomitantly decreasing miR-149 in hBMSCs.

By evaluating the H19, insulin-like growth factor 1 (IGF1), and miR-185-5p axis, whose members have been identified to have a regulatory function in the bone mineralization process, by using qPCR in MC3T3-E1 cells, Wu et al. [58] observed that expression levels of H19 and IGF1 are increased, while miR-185-5p is downregulated in mineralized osteoblasts compared to controls. Treatment either with H19 siRNA or miR-185-5p mimic inhibits the matrix mineralization of osteoblasts. Functionally, the results indicate that H19 could act as a ceRNA regulating IGF-1 expression via sponging miR-185-5p, thus promoting the mineralization in osteoblasts.

### 2.2. LncRNAs as Negative Regulators

In 2019, Chen et al. [59] found that lncRNA XIST was overexpressed in OP patients, by using Rank Prod analysis. Their studies reported that its overexpression inhibits osteoblast differentiation of BMSCs, as shown by the reduction of ALP activity and formation of hydroxyapatite crystals. Contrarily, cells treated with siRNA for lncRNA XIST show a recovery in ALP activity and bone mineralization.

After reports about the different roles of lncRNA XIST in several diseases and physiological cellular processes, including osteoblastic differentiation, Niu et al. [60] explored its possible role in fracture healing. Authors reported that the plasmatic levels of lncRNA XIST in fractured patients are upregulated with respect to those of the healthy group. It was then seen that MC3T3-E1 cells transfected with siRNA-XIST (si-XIST) showed lncRNA XIST downregulation and cell cycle-associated protein levels enhanced, compared to non-transfected cells. In addition, the lncRNA XIST downregulation increased ALP activity and attenuated the apoptosis process. They also discovered that this lncRNA targets miR-203-3p, which promotes osteogenesis and bone formation by regulating its gene target *ZFPM2* expression. Subsequently, *ZFPM2* expression was negatively correlated with miR-203-3p expression levels and positively with lncRNA XIST expression levels, suggesting that *ZFPM2* could inhibit osteoblast proliferation and differentiation, and could also be involved in the cellular apoptosis process. Further experiments also revealed that miR-203-3p overexpression can reverse *ZFPM2* effects. In conclusion, the authors demonstrated that lncRNA XIST downregulation promoted osteoblastogenesis and inhibited osteoblast programmed cell death, by acting through the miR-203-3p/ZFPM2 axis, suggesting that this molecular mechanism may represent a novel potential target for the treatment of fracture patients.

Shen et al. [61] studied the lncRNA HOTAIR function during osteogenic differentiation in hBMSCs. The authors found that its levels are higher in serum and hBMSCs isolated from OP patients compared to those in the control group. The use of hBMSCs transfected with sh-HOTAIR and HOTAIR overexpression vector permitted the authors to discover that a reduced expression of lncRNA HOTAIR increases ALP activity, matrix mineralization, and osteoblast marker gene expression levels. On the contrary, they observed that lncRNA HOTAIR overexpression leads to the opposite results. Moreover, the molecular mechanism of this lncRNA was investigated, finding that lncRNA HOTAIR was able to inhibit osteoblast differentiation through the Wnt/β-catenin signaling pathway suppression.

He et al. [62] identified that lncRNA RP11-527N22.2 significantly decreases during human umbilical cord mesenchymal stem cells (hUC-MSCs) osteogenic differentiation, by using Agilent Human lncRNA Microarray. Subsequently, this lncRNA was called osteogenic differentiation inhibitory regulator 1 (ODIR1), and its function was investigated. He et al. examined the role of lncRNA ODIR1 during osteogenic differentiation, discovering that its expression decreases during this process, while ALP activity and hydroxyapatite crystal formation increase. These observed data were then confirmed in vivo, in nude mice treated with ODIR1 vector, observing a reduction in bone formation with respect to untreated mice. Further studies highlighted that lncRNA ODIR1 can interact with F-box protein 25 (FBXO25), facilitating its degradation via proteasoma. On the contrary, lncRNA ODIR1 expression knockdown increases FBXO25, which promotes H2BK120 monoubiquitination and H3K4 trimethylation accordingly. Both of these modifications form a loose chromatin structure that promotes the transcription of several genes, among which *OSX*, a crucial osteogenic transcription factor.

The role of the lncRNA small nucleolar RNA host gene 1 (SNHG1) in osteogenesis was evaluated by Xiang et al. [63]. Based on previous studies about mir-101 and Dickkopf1 (DKK1) functions in osteogenesis, the authors decided to investigate their expression levels during hBMSC osteoblast induction by using qPCR. They found that miR-101 and osteogenic-related genes are upregulated, while DKK1 and lncRNA SNHG1 are downregulated during osteogenesis, suggesting that miR-101 overexpression could reduce DKK1 and lncRNA SNHG1 expression levels. Therefore, lncRNA SNHG1 could attenuate osteoblast differentiation by acting as a sponge of miR-101 and by inducing upregulation of *DKK1* expression levels. Finally, the authors reported for the first time the regulatory function of the new SNHG1/miR-101/DKK1 in the osteogenic differentiation process.

A study by Zhang et al. [64] explored the importance of lncRNA Urothelial Carcinoma Associated 1 (UCA1) into osteoblast proliferation and differentiation. First, the authors detected lncRNA UCA1 plasma levels in OP and healthy patients by using qPCR. Results revealed that UCA1 levels are higher in OP patients with respect to the healthy group, indicating that this lncRNA could play an important role in OP pathogenesis. Subsequently, they investigated the molecular mechanism of action of this lncRNA, and it was seen that lncRNA UCA1 inhibition in MC3T3E1 cells leads to an increase in osteoblast proliferation and differentiation. Furthermore, they discovered that the BPM2/Smad1/5/8 signaling pathway, which is very important for the osteogenesis process, is significantly activated after the inhibition of lncRNA UCA1.

Based on their previous studies about the role of lncRNAs in osteoblast differentiation, Yin et al. [65] identified the involvement of two other lncRNAs, AK039312 and AK079370, in the bone formation process. They found that the expression levels of both lncRNAs are highly expressed in OVX and aging mice, and are also correlated with osteogenesis marker gene expression in several age ranges of mice, suggesting that AK039312 and AK079370 lncRNAs could be involved in bone formation. Further studies have shown that these lncRNAs are capable of inhibiting two crucial transcription factors of the Wnt/β-catenin signaling pathway, TGF7 and LEF1, by preventing osteogenesis accordingly. Moreover, the authors demonstrated that these lncRNAs could act as a sponge of miR-199b-5p by regulating its downstream target GSK-3β, a Wnt/β-catenin signaling pathway inhibitor. The subsequent analysis also proved that lncRNA AK039312 and AK079370 combined activity is more effective than the action of the single lncRNAs. Therefore, in this study, Yin et al. showed a potential novel therapeutic approach based on the simultaneous use of different siRNAs against lncRNAs with similar functions.

Jiang et al. [66] analyzed the serum expression of DANCR between 44 fractured patients and 24 healthy controls (HC) by using RT-PCR. The results obtained showed that the expression levels of this lncRNA are significantly higher in patients with fractures compared to HC. Subsequently, the MC3T3-E1 cell in vitro model was used to explore the effect and the molecular mechanisms of DANCR knockout on MC3T3-E1 cell proliferation and differentiation. Based on the results of in vitro experiments, DANCR inhibition could promote the proliferation and differentiation of MC3T3-E1 cells through the upregulation of the Wnt/β-catenin signaling pathway.

The aim of a study by Cai et al. [67] was to investigate the effect of ANCR on the proliferation, ALP activity, calcium nodules, and apoptosis in osteoblast cells derived from postmenopausal OP (PMOP) mouse models. Compared with the control group, the expression levels of ANCR and intracellular calcium concentration are remarkably higher in the PMOP mouse group, while experiments performed on cells transfected with siRNA-ANCR show that ANCR silencing facilitated the proliferation and osteogenic differentiation process. This lncRNA has been reported to be capable of downregulating RUNX2 expression by specific binding to the enhancer of zeste homolog 2 (EZH2).

AK045490 has previously been shown to be a possible osteoblastic differentiation inhibiting lncRNA. For this reason, Li et al. [68] selected this lncRNA to confirm its role in osteoblast differentiation in vitro and in vivo. In vivo experiments carried out on two osteoporotic mouse models showed that its expression levels are significantly higher in the OVX mice and age-related osteoporotic groups compared, respectively, to sham-operated (Sham) and young groups, indicating that bone loss and bone structural deterioration are associated with elevated AK045490 expression levels. Subsequently, in vitro analysis in MC3T3-E1 cells transfected with AK045490 siRNA or NC siRNA showed that this lncRNA plays a negative regulatory role in the differentiation of osteoblasts, as revealed by the significant increase of the relative osteogenic marker gene expression, ALP activity, and the formation of mineralized nodules in the AK045490 siRNA group. Further in vitro analyses were performed to elucidate the mechanism of the inhibition function of AK045490 on osteoblast differentiation by studying the *β*-catenin/TCF1/RUNX2 pathway. Based on the results obtained, AK045490 prevents the nuclear translocation of β-catenin via the downregulation of TCF1, LEF1, and RUNX2 expression. By bioinformatic analysis, they supposed that AK045490 functions as a ceRNA, potentially by targeting bone associated-miRNAs, such as miR-6344, miR-3089, and miR-6951. Finally, in vivo administration of AK045490 siRNA enhances bone formation in an OVX mouse model.

Li et al. [69] detected the lncRNA maternally expressed 3 (MEG3) in the serum of fractured patients and explored its possible mechanism of action during osteoblast proliferation and differentiation. It was seen that lncRNA MEG3 levels were higher in the serum of fracture patients compared to those of healthy patients, indicating its possible participation in fracture healing. Subsequent studies revealed that MC3T3-E1 cells treated with MEG3 siRNA show a decrease of lncRNA MEG3 expression levels, while osteoblast proliferation and differentiation increase. Given the fundamental action of the Wnt/β-catenin signaling pathway on bone formation, the authors investigated the relationship between this pathway and lncRNA MEG3 expression, discovering that cells treated with MEG3 siRNA show an increase of Wnt/β-catenin signaling pathway.

In the last of three studies on the role of lncRNA H19 in osteogenesis, Xiaoling et al. [70] investigated the serum expression levels of miR-19b-3p and lncRNA H19 between 18 osteoporotic patients and 12 HC, and their effects on osteoblast proliferation and differentiation of BMP-2-induced hBMSCs. Serum expression levels of miR-19b-3p resulted to be elevated, while those of H19 resulted to be downregulated in osteoporotic patients compared to the control group. Experiments on BMP-2-induced hBMSCs transfected with miR-19b-3p mimic or inhibitor and pcDNA3.1-H19 or pcDNA3.1-H19 + miR-19b-3p mimic revealed that miR-19b-3p promotes hBMSC proliferation and differentiation, while H19 inhibits both these processes, through sponging miR-19b-3p. In addition, hBMSCs exposed to different concentrations of 17β-Estradiol (E2) demonstrated that H19 and miR-19b-3p expression levels are inversely correlated in a dose-dependent manner.

### 2.3. Bioinformatic Study

Hong et al. [71] investigated the lncRNA-mRNA crosstalk, by using computational analysis, to realize a network called “osteoblast-differentiation-associated lncRNA-mRNA network” (ODLMN). First, the authors identified lncRNAs and genes differentially expressed in osteogenic differentiation. Among 14.000 genes and 4.000 lncRNAs characterized, they found that 1.017 genes and 662 lncRNAs are differentially expressed in human mesenchymal stem cells during osteoblast differentiation compared to control cells, suggesting that the identified lncRNAs could represent potential regulators of the osteogenic differentiation process. Second, they performed many topology analyses for ODLMN, identifying several lncRNAs with central topology structures, which could be involved in several osteoblast differentiation regulating key pathways. Third, they found two functional modules containing lncRNA-mRNA interaction highly related to osteogenic differentiation by using the “MCODE” clustering algorithm for OLDMN. Therefore, these results suggested that lncRNAs may act synergistically with mRNAs in osteoblast differentiation. Moreover, the authors saw that lncRNAs could also synergize with transcription factors to regulate several biologic processes in osteogenic differentiation. In summary, the ODLMN construction by Hong et al. demonstrated that lncRNA-mRNA crosstalk may play an important role in osteogenesis, and different functional lncRNAs could be key molecular regulators in the osteogenic differentiation process.

Taken together, these results, which have been described by analyzing several recent studies on the role of lncRNAs on osteogenesis (Table 1), suggest that the exploration of lncRNAs and their related functional mechanisms could lead to the development of novel diagnosis and treatment strategies for bone metabolism-related diseases, in which the osteogenesis process is altered.

## 3. LncRNAs That Regulate Osteoclastogenesis

### 3.1. LncRNAs as Positive Regulators

Little is known about the epigenetic regulation of osteoclast biology and functionality, but some of the studies reported below investigate the roles of lncRNAs during osteoclastogenesis.

Based on previous studies, endothelial progenitor cells (EPCs) were shown to boost migration and osteoclast differentiation of bone marrow-derived macrophages (BMMs), both in vitro and in vivo.

Cui et al. [72] assessed whether EPC-derived exosomes could stimulate osteoclastogenesis through the MALAT-1/miR-124 axis. Exosomes released from EPCs promote the migration and osteoclast differentiation of co-cultured BMMs by increasing the expression of MALAT1 and Integrin subunit β 1 (ITGB1) and by reducing that of miR-124. Bioinformatics prediction, dual-luciferase reporter assays, and in vitro experiments showed that MALAT1 in EPC-derived exosomes negatively regulates miR-124, enhancing ITGB1, as well as osteoclast differentiation marker genes (i.e., MMP9, CTSK, TRAP, and CAR2), expression levels. A mouse fractured model co-transplanted with EPC-derived exosomes and BMMs exhibited major neovascularization at the side of the fracture and an enhanced fracture healing compared to those treated with BMMs alone, partly due to the sponging function of MALAT1 against miR-124. In this way, Cui et al. reported how EPC-derived exosomes could promote bone repair in vivo and, therefore, have a positive influence on osteogenic induction in a bioactive scaffold.

Zhang et al. [73] investigated the effect and the expression of DANCR, miR-34a-5p, and Jagged1, previously found implicated in bone remodeling [74,75,76,77], on the osteoclastogenesis and root resorption of PDL cells treated with compression force (CF) and in a rat orthodontic tooth movement (OTM) model. The expression levels of DANCR, Jagged 1, and RANKL are significantly upregulated, while those of miR-34a-5p have an opposite trend in the rat OTM model. The same expression pattern was observed in vitro in a time-dependent manner with a peak at 24h. Bioinformatic and luciferase reporter assay revealed that the expression of DANCR and miR-34a-5p is negatively correlated in HEK293 cells. Moreover, in vitro experiments indicate that CF treatment promoted osteoclastogenesis and root resorption via Jagged1 through the regulation of DANCR and miR-34a-5p expression. Finally, in vivo analysis showed that DANCR expression, Jagged1, and RANKL protein levels are decreased after DANCR silencing compared with the control group, suggesting that the knockdown of DANCR reduced the osteoclastogenetic effects induced by CF and, therefore, could be a potential therapeutic target for orthodontically induced inflammatory root resorption (OIIRR).

Recently, expression levels of lncRNA nuclear paraspeckle assembly transcript 1 (NEAT1) were found deregulated between osteoporotic patients compared to HC, but its molecular mechanism in bone metabolism remains unclear. For this reason, Zhang et al. [78] explored the effects of NEAT1 on osteoclastogenesis and dissected its underlying regulatory mechanisms in OP. NEAT1 expression was found upregulated during osteoclastogenesis induced by RANKL in a dose-dependent manner. The expression of osteoclastic-related marker genes, such as a *nuclear factor of activated T cell c1* (*NFATc1*), *integrin β3*, *TRAP5*, *DC-STAMP*, and *cathepsin K*, is respectively increased and reduced in BMM cells transfected with recombinant lentivirus coding for NEAT1 and shRNA against this gene. Mechanistically, NEAT1 interacted with miR-7, thereby interfering with miR-7-mediated control of PTK2. In vivo experiments revealed that NEAT1 overexpression prompts osteoclastogenesis and induces bone loss, suggesting its potential as an osteoporotic therapeutic target.

Du et al. [79] aimed to clarify the influence of lncRNA TUG1 on osteoclast differentiation. The obtained results show an upregulation of the TUG1 expression levels following M-CSF + RANKL-induced osteoclast differentiation in CD14^+^ PBMCs, while TUG1 silencing inhibits osteoclastogenesis, as evidenced by the reduction of TRAP-positive osteoclasts and by low protein levels of TRAP, NFATc1, and osteoclast-associated receptor (OSCAR). At the same time, the obtained data reveal an increase of V-maf musculoaponeurotic fibrosarcoma oncogene homolog B (V-MafB) expression level. Functional in vitro experiments through the TUG1 upregulation and downregulation revealed that TUG1 stimulates osteoclast formation by modulating MafB and, in particular, by facilitating its degradation. Likewise, in vivo evidence confirmed that TUG1 overexpression increases the velocity of OTM and bone resorption by promoting osteoclastogenesis in rats.

Ling et al. [80] investigated the expression levels and effects of lncRNA MIRG on the osteoclast formation of BMMs isolated from mouse femur. qPCR analysis results showed that both expression levels of MIRG and NFATC1 are markedly increased in BMM-derived osteoclasts compared to controls. After MIRG silencing through lentiviral transfection, a downregulation of marker genes associated with osteoclastic differentiation and bone resorption, including NFATC1, TRAP, c-FOS, CTSK, MMP9, and DC-STAMP, is detected. Using bioinformatics prediction tools, miR-1897 is one of the miRNA targets of MIRG, and its expression is decreased and negatively correlated with that of MIRG. Luciferase reporter assay demonstrated that MIRG downregulated the expression of miR-1897 via direct binding, thereby also controlling the NFATC1 expression in vitro and in vivo. Overall, lncRNA MIRG could stimulate osteoclastogenesis and bone resorption by acting as a molecular sponge against miR-1897.

### 3.2. LncRNAs as Negative Regulators

The aim of a study by Chen et al. [81] was to investigate the influence of lncRNA Bmncr, previously established to promote osteogenesis and adipogenesis, in NF-kB receptor activator (RANKL)-induced osteoclast differentiation. qPCR results revealed that Bmncr expression declines over time, reaching its lowest levels at 72h during RANKL-induced osteoclast differentiation. RAW 264.7 cells, transfected with lentivirus containing Bmncr, have reduced TRAP-positive cells, lower bone resorption capacity, and downregulated osteoclast-related marker genes (i.e., *Atp6v0d2*, *Acp5*, *Ctr*, and *Mmp9*) expression levels, whereas these effects were reversed after its silencing. The authors also reported a lower Bmncr level in spleen and marrow tissue of OVX mice compared with controls.

Zhang et al. [82] in their study clarified the effect of lncRNA repressor of the nuclear factor of activated T cells (NRON) on osteoclastogenesis during OTM. They performed qPCR analyses to compare its expression in human alveolar bone obtained respectively from young and elderly individuals during wisdom tooth removal and in an orthodontic mouse model compared with wild-type mice. The results showed that NRON expression is downregulated during osteoclast formation and bone aging. The Nron overexpression in osteoclastic NRON transgenic mice suppresses the orthodontic movement of teeth, the number of TRAP-positive cells, as well as the expression pattern associated with bone resorption markers (i.e., Trap, Dcstamp, Mmp9, and NFATC1). The inhibitor effect of NRON on osteoclast formation is caused by preventing nuclear translocation of NFATC1. Conversely, when osteoclastic NRON knockout (Nron CKO) mice are generated, they present an accelerated OTM. In relation to these, Zhang et al. concluded that lncRNAs could play a critical role in regulating OTM throughout orthodontic treatment.

### 3.3. Bioinformatics Studies

Li et al. [83] compared the gene expression profile in peripheral blood monocytes between high versus low bone mineral density (BMD) individuals obtained from the Gene Expression Omnibus (GEO) database, to identify differentially expressed genes according to the *p*-value < 0.05 and fold change. Results from this analysis revealed 496 differentially expressed genes in low BMD subjects (376 downregulated and 120 upregulated). Among these, there are four zinc finger genes (i.e., *ZNF79*, *ZNF223*, *ZNF528*, and *ZNF765*) and 24 differentially expressed lncRNAs. Function enrichment analysis revealed that upregulated and downregulated genes are mainly enriched in the inflammation-associated pathway and pathways regulating bone mineral density, respectively. The downregulated ones (i.e., HDAC 4 and 5, JDP2, BRD4, ACSL1, ACADVL, CDYL2, CNR1, CCL25, HCAR1, HCAR2, GNG13, and GNG8) are hub proteins in the PPI network and are reported to play a key role in bone mineral homeostasis. In addition, 4 upregulated proteins (i.e., MEX3C, TRIM4, MKRN1, and MYLIP) are hub genes/proteins in the upregulated PPI network. Finally, lncRNA, RP11-498C9.17 is principally enriched in the epigenetic regulation pathway; therefore, this lncRNA could act as a key epigenetic factor in bone mineral homeostasis and, in particular, on osteoclastogenesis.

Liu et al. [84] investigated lncRNA and mRNA expression patterns during osteoclast differentiation of CD14^+^ monocytes. Among the 17.246 lncRNAs and 111.132 mRNAs analyzed by using Illumina RNA sequencing, 10 lncRNAs, of which 9 downregulated and 1 upregulated, and 10 mRNAs, of which 7 upregulated and 3 downregulated, were significantly differentially expressed after osteoclast differentiation in the qPCR-based validation step. Functional enrichment analysis revealed 2.442, 280, and 374 differentially expressed gene ontology terms respectively in Biological Process, Cellular Component, and Molecular Function in osteoclast differentiation, and Kyoto Encyclopedia of Genes and Genomes (KEGG) analysis indicated that most signaling pathways are significantly enriched in those with a pivotal role in osteoclast differentiation (i.e., PI3K-AKT, MAPK, and NF-kB). Furthermore, co-expressing and ceRNA networks demonstrated that the ENSG00000257764.2/miR-106a-5p/TIMP2 axis could act as a regulatory mechanism of osteoclast differentiation.

Therefore, as reported before for lncRNA and its role in osteogenesis from the analyses of the few recent studies on their role on osteoclast differentiation (Table 2), we observed also here that these molecules could represent a future diagnostic and therapeutic target for the treatment of bone resorption.

## 4. LncRNAs in Primary Bone Tumors and OP

### 4.1. LncRNA GAS5

*GAS5* is a tumor suppressor gene located at chromosome 1q25.1 comprised of 12 exons non-encoding proteins, whose highly conserved introns encode an important lncRNA involved in carcinogenesis and tumor progression, marked as lncRNA GAS5 [85]. In fact, its expression was found to decrease in cancer tissues and cells compared with normal tissues and cells in several cancers, including OS [85]. Functional studies have shown that lncRNA GAS5 silencing significantly enhanced cell viability, migration, invasion, and apoptosis on in vitro models for studying OS, as opposed to what happens following lncRNA GAS5 overexpression.

Furthermore, this deregulated expression has been significantly correlated with clinicopathological characteristics (i.e., tumor staging, size and histological grading, and the formation of distant metastasis) and prognosis [85].

Although the exact molecular mechanism of action of lncRNA GAS5 in OS is far from being understood, several studies have proposed that it would act mainly as a ceRNA, thus regulating several signaling pathways frequently associated with cancer progression. In particular, lncRNA GAS5 has been reported to interact with miR-23a-3p, miR-203a, miR-221, and miR-663a.

It has been observed that GAS5 overexpression could block OS cell proliferation and invasion via regulation of miR-23a-3p/phosphatase and tensin homolog (PTEN)/Phosphoinositide 3-kinase(PI3K)/AKT pathway [86]. In addition, Wang et al. described that the observed inhibition of OS cell proliferation and progression is the result of the interaction of lncRNA GAS5 with miR-203a, which is at the base of the prevention of the interaction between miR-203a and TIMP Metallopeptidase Inhibitor 2 (TIMP2), resulting in deactivation and activation, respectively, of PI3K/AKT/GSK3β signaling and NF-κB signaling [87]. Ye et al. have also reported that GAS5 could suppress cell growth, migration, and epithelial–mesenchymal transition (EMT) in OS by modulating the miR-221/aplasia Ras homolog member I (ARHI) axis [88]. At the same time, Yao et al. observed that GAS5 could also stimulate OS progression suppressing Ras Homolog Family Member B (RHOB) expression through sponging miR-663a [89].

Furthermore, GAS5 could function as a ceRNA sponging of miR-663a to regulate the expression levels of downstream target *MYL9* gene and thus suppress proliferation and migration of OS cells [90].

In addition, some studies have revealed that lncRNA GAS5 could control osteogenic and adipogenic fates of hBMSCs [45,91,92]. However, its role and the effect on osteogenesis, and therefore its potential application as a diagnostic and therapeutic strategy against OP, need to be clarified.

Recently, two different studies revealed that lncRNA GAS5 could be a positive regulator of osteoblast differentiation as evidenced by the ALP activity of human multipotent cells derived from osteoporotic patients. Functionally, in the first study [93], the authors proposed that GAS5 significantly increases RUNX2 expression through regulating miR-498, thereby inhibiting the progression of OP. At the same time, Li et al. [91] also found that lncRNA GAS5 expression was downregulated in bone tissue and cells derived from osteoporotic patients and that its adenovirus-mediated overexpression promoted osteogenesis both in vitro and in vivo via the regulation of UPF1/SMAD7 axis.

Other studies investigated the expression profile of circulating lncRNA GAS5 between osteoporotic patients and HCs. Both these studies found that its expression levels were significantly increased, respectively, in plasma and serum of osteoporotic patients compared to the control group [94,95]. In addition, Cong et al. [95] found that miR-21 had an opposite expression pattern compared to lncRNA GAS5 in the plasma of osteoporotic patients, and in vitro experiments carried out in osteoclast transfected with this lncRNA showed that its overexpression induces their apoptosis through the downregulation of miR-21. Based on these findings, circulating lncRNA GAS5 could be considered a potential biomarker for the diagnosis and prognosis of OP.

No studies have analyzed lncRNA GAS5 expression in ES specimens.

Table 3 reports the evidence on the role of lncRNA GAS5 in OS and OP.

### 4.2. LncRNA TUG1

TUG1 is a lncRNA with a length of 7.1 kilobases (kb) located at chromosome 22q12. It was first identified during an upregulated genes screening in developing mouse retinal cells upon treatment with taurine [96]. Its expression has been demonstrated deregulated in a wide range of common human cancers. in particular, it has been reported to be upregulated in esophageal cancer [97], hepatocellular carcinoma [98], urothelial bladder cancer [99], and ovarian cancer [100], where it promotes cell proliferation, migration, and invasion, acting as an oncogenic lncRNA. It has also been found to be downregulated in non-small cell lung cancer (NSCLC) and in multiple myeloma (MM) [101,102]. Although TUG1 has been investigated in several types of tumors, it has only recently been studied in OS, in the primary bone tumor, as well as other bone metabolic disorders.

Over recent years, TUG1 overexpression has also been described in OS cells and tissues regarding adjacent non-tumor tissue and normal osteoblast cells. It has also been observed that its upregulation is meaningfully correlated with poor prognosis and distant metastases, as well as clinical status. On the other hand, TUG1 silencing inhibits the proliferation and invasion of OS cells [103].

Several studies have demonstrated that TUG1 could promote OS tumorigenesis by acting as a ceRNA negatively regulating miRNA expression through their sponging.

Wang et al. demonstrated that TUG1 contributes to OS progression by epigenetically regulating miR-153 expression [104].

Yu et al. revealed that TUG1 functions as a ceRNA of miR-143-5p, abrogating miRNA-dependent suppressive effects on HIF-1α expression and, as such, this axis controls cellular proliferation, metastasis, and angiogenesis, both in vitro and in vivo [105].

Xie et al. [106] reported that TUG1 was able to function as an endogenous sponge of miR-9-5p, reversing its effect on the proliferation, cell cycle arrest, colony formation, and apoptosis, and on the downregulation of POU class 2 homeobox 1 (POU2F1) expression, thus potentially facilitating the onset and progression of OS.

In a study by Wang et al., Rho-associated coiled-coil protein kinase 1 (ROCK1) expression and ROCK1-mediated migration and invasion were positively affected in human OS cells overexpressing TUG1 through sponging of miR-335-5p [107].

Li et al. provided evidence about the critical role of TUG1 in promoting OS cell proliferation and invasion by inhibiting miR-212-3p expression [108].

Li et al. showed that TUG1 facilitates proliferation and represses apoptosis by influencing the expression levels of miR-132-3p and SRY-related HMG-box (SOX4) in human OS cell lines and primary OS cells [109].

The results of a study by Zhao et al. [110] show that TUG1 could serve as a molecular sponge of miR-140-5p to abolish its repressive effect on Profilin 2 (PFN2), thus markedly increasing cell proliferation, migration, invasion capacity in human OS cell lines and, in addition to TUG1 silencing, could decrease tumor growth in OS xenograft models.

Cao et al. [111] indicated that TUG1 plays an important role in OS progression through the activation of the Wnt/β-catenin pathway mediated by EZH2 by sponging miRNA-144-3p.

Collectively, the above-cited studies show that TUG1 could play an oncogenic role in OS by functioning as an miRNA sponge, providing a novel possible therapeutic target for patients suffering from OS.

In another study, Zhou et al. [112] observed that TUG1 knockdown in cancer cells resistant to the development of cisplatin (DDP) suppressed their cell growth and increased apoptotic rate, possibly through modulating MET/AKT signaling pathway.

Han et al. [113] reported that the effect of TUG1 on glycolysis, through the regulation of the hexokinase-2 (HK2), the first rate-limiting enzyme in this process, could be a key mechanism by which it negatively affects the viability of OS cells. Yun-Bo et al. [114] described that TUG1 upregulation in U2OS cells results in increased cell proliferation compared with a normal cell line, probably acting partially via the regulation of the AKT signaling pathway.

Finally, data from a study by Sheng et al. [115] provides evidence about the oncogenic role of TUG in promoting OS development through the activation of the RUNX2 pathway.

Overall, these findings provide new evidence about the oncogenic role and novel thoughts about the molecular mechanisms of TUG1 in promoting the development of OS, suggesting this lncRNA as a potential novel biomarker for improving diagnosis and prognosis of OS.

No studies have analyzed lncRNA TUG1 expression in ES and OP specimens.

Table 4 reports evidence on lncRNA TUG1 in OS.

### 4.3. LncRNA MALAT1

MALAT1 is a lncRNA of 8.5 kb, located at 11q13. It has been shown that this lncRNA is largely expressed in normal tissues and conserved among other mammalian species, indicating its possible important function during development and evolution. Furthermore, several studies have revealed the contribution of lncRNA MALAT1 in cancer development and progression by modulating different signaling pathways. It has also been seen that its aberrant expression in body fluids and/or tumor tissues could represent a possible diagnostic and/or prognostic biomarker for several types of tumors, including OS [116]. Recent studies have demonstrated that lncRNA MALAT1 can promote OS development and progression by acting as a sponge for several miRNAs and modulating the downstream pathways.

Wang et al. [117] demonstrated that MALAT1 can promote OS cell proliferation and migration by directly sponging miR-26a-5p. Furthermore, they revealed that the transcriptional factor FOXO1 negatively regulated MALAT1, suggesting it as a possible therapy for OS treatment.

A study conducted by Duan et al. [118] led to the understanding that MALAT1 supports OS cell viability, invasion, and migration by acting as a ceRNA, inhibiting miR-34a expression and upregulating its target gene, cyclin D1 (CCND1).

Zhang et al. [119] showed that lung metastases of OS were stimulated by the action of MALAT1 to sponge miR-202. Chen et al. [120] reported that the oncogenic function of MALAT1 was explicated in OS through the increase of stem cell-like properties, by sponging miR-129-5p, thus augmenting its downstream RET-Akt pathway.

Data collected by Wang et al. [121] suggest that MALAT1 acts as a sponge for mir-144-3p, upregulating ROCK1/ROCK2 expression and inducing OS proliferation and metastasis.

Li et al. [122] showed that overexpression of MALAT1 increase OS growth and progression through suppression of miR-205 and activation of its gene target, *SMAD4*.

Sun et al. [123] illustrated the role of MALAT1 in OS, reporting that this lncRNA can promote cell proliferation in OS by regulating HDAC4 expression by decoying miR-140-5p.

Liu et al. [124] indicated that MALAT1 functions as a ceRNA by sponging miR-142-3p and miR-129-5p and upregulating the downstream factor high-mobility group protein B1 (HMGB1), subsequently enhancing cell proliferation and tumor progression.

Another study by Luo et al. [125] showed that the growth of OS cells increases via the sponging effect of MALAT1 on miR-376a, resulting in upregulation of TGFα expression levels.

Liu et al. [126] reported that MALAT1 raises TGIF2 expression levels through the negative regulation of miR-129-5p, which promotes the proliferation, invasion, and migration of OS cells.

Zhang et al. [127] identified a new axis composed of MALAT1/miR-509/Rac1 that could be involved in OS growth and metastasis. In fact, MALAT1 acts as a ceRNA to hinder miR-509 and activate the RAC1/JNK pathway, thus promoting OS cell proliferation and tumor progression.

Several studies have demonstrated that lncRNA MALAT1 promotes OS growth and progression through other mechanisms, not involving only its miRNAs sponging function as reported before.

Cai et al. [128] observed that the knockdown of MALAT1 in several OS cell models led to cell proliferation and migration inhibition, while cell cycle arrest and apoptosis were promoted. Moreover, they saw that MALAT1 could support OS development through the regulation of the RhoA/ROCK pathway. Furthermore, two studies understood that MALAT1 supports OS metastasis through its interaction with EZH2, resulting in a downregulation of E-cadherin expression [129] and enhancing the β-catenin signaling pathway [130]. In addition, Dong et al. [131] discovered that MALAT1 knockdown leads to PI3K/Akt pathway inactivation, suggesting that the proliferation and metastasis of OS are stimulated by this lncRNA through the activation of the PI3K/Akt signaling pathway. Finally, Zhang et al. [132] confirmed previous evidence about the pro-angiogenetic effect of MALAT1 on OS progression, via a mechanism involving the target of rapamycin (mTOR) and hypoxia-inducible factor-1α (HIF-1α).

Regarding another common primary bone tumor, Ewing’s Sarcoma (ES), only a few studies have shown that expression levels of lncRNA MALAT1 are upregulated in ES cells and tissues. Although its molecular mechanism is unclear, it has been shown that this lncRNA could be involved in ES development.

In particular, Sun et al. [133] discovered that Spleen tyrosine kinase (SYK) increased the transcription of MALAT1 by regulating c-MYC oncogene. Moreover, a study carried out by He et al. [134] revealed that the overexpression of MALAT1 could be due to the activation of Tenascin-C (TNC)/Integrin α5β1/YAP axis, thus promoting the ES progression.

Since several studies have shown that many lncRNAs could play a crucial role in bone turnover-associated processes, assuming that deregulation of their expression profile could be correlated with the occurrence of OP, MALAT1 could also play a role in OP.

Hong et al. [135] identified two possible networks of ceRNAs involved in the pathogenesis of OP according to the outcomes obtained from bioinformatics programs, such as NEAT1/MALAT1-hsa-miR-22-3pPTEN/ESR1/ERBB3/CSF1R/CDK6 and NEAT1/MALAT1-hsa-miR-32-3p-SP1/FZD6 axis, which could have the potential to be used as target molecules for the future development of therapy against OP.

A study conducted by Zheng et al. [136] reported the role of MALAT1 in the onset of OP, describing that the downregulation of this lncRNA inhibited osteogenic differentiation of BMSCs by promoting the activation of the MAPK signaling pathway and, consequently, the progression of this disease.

Based on previous studies about the communication between osteoblasts and osteoclasts through the release of exosomes and the role of lncRNA MALAT1 and miR-34c in bone homeostasis, Yang et al. [137] tried to clarify the possible function of exosomes containing lncRNA MALAT1 derived from BMSCs in OP. They found that BMSC-derived exosomal MALAT1 could further promote bone formation and mitigate the symptoms of OP by acting as a molecular sponge for mir-34c, therefore increasing the expression of SATB2.

Table 5 reports evidence about lncRNA MALAT1 in OS, ES, and OP.

## 5. Discussion

As a result of recent advances in high-throughput sequencing analysis, ncRNAs have gained the attention of the scientific community, especially in the field of personalized medicine. In the pre-genomic era, ncRNAs were considered to have an insignificant role in cell biology, but increasing findings show that they have a huge influence on several processes, thus revealing a new level of regulation in the more complex organisms.

ncRNAs belong to epigenetic control mechanisms, which are heritable phenotypic modifications, not attributable to changes in DNA sequences.

Recently, lncRNAs, a subclass of ncRNAs longer than 200 nucleotides, have drawn attention as essential regulators of several cellular processes to maintain homeostasis. Their expression pattern has been demonstrated to correlate strictly between physiological and pathological conditions, and they are therefore candidates for innovative molecular approaches in potential diagnostic, prognostic, and therapeutic biomarkers for a wide range of diseases, including cancer [138,139].

A biomarker has been defined as “any substance, structure, process or its products that can be measured in the body and that impacts or foresees the incidence of outcome or disease” by the World Health Organization (WHO), in conjunction with the International Labor Organization and the United Nations [140].

Bone undergoes a continuing, balanced osteoclast- and osteoblast-mediated process of resorption and formation, required for the preservation of bone homeostasis throughout life. In normal conditions, bone mass is maintained, resulting in a mature bone structure and maintenance of homeostasis of the systemic mineralized metabolism. On the other hand, an impaired equilibrium of the bone remodeling process may result in bone mass loss or excessive bone deposition, leading to the occurrence of pathological conditions affecting the bone [141,142].

Recently, studies concerning the effects of lncRNAs in regulating bone metabolism have been carried out [28], although the understanding of the molecular mechanisms by which these molecules could affect the osteoblastogenesis and osteoclastogenesis processes is still at the beginning.

The interaction between lncRNAs and miRNAs is an emerging area of interest. As described in this review, several studies have shown that lncRNAs could play their role as regulators of gene expression, for example, by acting as a ceRNA via direct binding with miRNAs of interest [28,143]. Therefore, future investigations aimed at increasing knowledge about the molecular interactions between lncRNAs, miRNAs, and mRNAs could help discover new complex networks, to develop ncRNA-based therapeutic strategies against bone-related disorders.

However, to date, there are no molecular strategy drugs available in clinical trials able to modulate lncRNA expression in vivo. The design of specific lncRNA-based therapies that antagonize the expression levels and the effects of oncogenic lncRNAs, or that increase the expression levels and the effects of oncosuppressor lncRNAs, could have a future as RNA-based therapeutic approaches for the treating of various bone-related diseases [139].

One of the major challenges of their potential applications in clinical practice is the few sequence similarities among species, because findings established in animal models may not be directly transferable to humans.

In recent years, in addition to cellular lncRNAs, a promising area of research has been the investigation of the functions of circulating lncRNAs (c-lncRNAs) [14]. In fact, lncRNAs can be detected in biological fluids, such as serum, plasma, whole blood, and urine, suggesting their potential as novel circulating biomarkers. Despite this, as discussed in this review, there are still very few studies on the evaluation of their expression levels in extracellular biofluids. As reported by Hackl et al. [144] for the identification of circulating miRNAs as diagnostic, prognostic, staging, and therapeutic biomarkers, an initial systematic investigation and a subsequent validation of candidate lncRNAs could be performed to identify a specific c-lncRNA signature that might be used for the management of patients affected by the metabolic bone disorder, but also virtually for all diseases.

In summary, this review gives an overview of the potential lncRNAs that may be innovative molecular biomarkers, not only to help with earlier diagnosis, prior to the occurrence of three particular bone metabolism-related disorders, such as OP, OS, and EWS, to direct the patients towards early treatment, thus impacting positively on care, but also for the identification of novel specific target molecules, which are essential for the development of RNA-based strategies against disorders associated with bone turnover.

Future studies aimed at increasing knowledge about the role of lncRNAs, as well as establishing more effective approaches regarding lncRNA expression levels, are needed to translate these potential biomarkers into clinical practice as personalized medicine.

## Figures and Tables

**Table 1:** Involvement of lncRNAs in osteoblastogenesis.

Sources	lncRNAs	Regulation Mechanism	Potential Role in Osteoblastogenesis	Study
Cells	PCAT1	Sponging miR-106a-5p (BMP2)	Regulates osteogenic differentiation	[34]
Cells	PGC1β-OT1	Sponging miR-148a-3p (KDM6B)	Regulates osteogenic differentiation	[35]
Cells and animals	OGRU	Sponging miR-320-3p (HOXA10)	Regulates bone formation	[36]
Animals	AK023948	Regulates PI3K/AKT pathway	Role in OP occurrence and development	[37]
Cells	LINC02349	Sponging miR-25-3p and miR-33b-5p (SMAD5 and Wnt10b)	Regulates osteogenic differentiation	[38]
Cells	KCNQ10T1	Sponging miR-701-3p (FGFR3)	Regulates osteoblast survival, migration, and proliferation	[39]
Cells	KCNQ10T1	Sponging miR-214 (BMP2)	Regulates osteoblast differentiation	[40]
Cells	LOC100506178	Sponging miR-214-5p (BMP2)	Regulates osteoblast differentiation	[41]
Cells	LINC00707	Sponging miR-370-3p (Wnt2B)	Regulates osteogenic differentiation	[42]
Cells	TUG1	Regulates Wnt/β-catenin pathway	Regulates bone formation	[43]
Cells	TUG1	Sponging miR-545-3p (CNR2)	Regulates osteoblast proliferation, and differentiation	[44]
Cells	GAS5	Sponging miR-135a-3p (FOXO1)	Regulates osteoblast differentiation	[45]
Cells	GAS5	Regulates MAPK signaling pathway	Regulates osteogenic differentiation	[46]
Cells	MALAT1	Sponging miR-30 (RUNX2)	Regulates osteogenic differentiation	[47]
Tissue and cells	DANCR	Regulates NF-kB pathway (ALP and RUNX2)	Regulates osteogenic differentiation	[48]
Cells and animals	Rhno1	Sponging miR-6979-5p (BMP2)	Regulates osteoblast differentiation	[49]
Cells and animals	Crnde	Regulates Wnt/β-catenin pathway	Regulates osteoblast proliferation and differentiation	[50]
Tissue, cells and animals	lnc-ob1	Regulates OSX expression	Regulates osteoblast differentiation	[51]
Cells	lnc-SUPT3H-1:16	Sponging miR-6797-5p (RUNX2)	Regulates osteoblast differentiation	[52]
Cells and animals	ODSM	Sponging miR-139-3p (ELK1)	Regulates bone formation	[53]
Tissue and cells	SNHG7	Sponging miR-9 (TGFBR2, p-smad2 and p-smad3)	Regulates osteoblast activity, migration, and proliferation	[54]
Cells	LINC00341 and PACERR	Regulate osteoblast specific marker genes	Role in OP occurrence	[55]
Cells	MCF2L-AS1	Sponging miR-33a (RUNX2)	Regulates osteogenic differentiation	[56]
Cells	H19	Sponging miR-149 (SDF-1)	Regulates osteoblast differentiation	[57]
Cells	H19	Sponging miR-185-5p (IGF-1)	Regulates the mineralization in osteoblasts	[58]
Cells	XIST	/	Regulates OP occurrence	[59]
Cells and plasma	XIST	Sponging miR-203-3p (ZFPM2)	Regulates osteoblast proliferation and differentiation	[60]
Cells and serum	HOTAIR	Regulates Wnt/β-catenin pathway	Regulates osteoblast differentiation	[61]
Cells	ODIR1	Regulates OSX expression	Regulates osteogenic differentiation	[62]
Cells	SNHG1	Sponging miR-101 (DKK1)	Regulates osteogenic differentiation	[63]
Cells and plasma	UCA1	Regulates BMP2/Smad1/5/8 pathway	Regulates osteoblast proliferation and differentiation	[64]
Cells and animals	AK079370 and AK039312	Sponging miR-199b-5p (Wnt/β-catenin pathway)	Regulate bone formation	[65]
Cells and Serum	DANCR	Regulates Wnt/β-catenin pathway	Regulates osteoblast proliferation and differentiation	[66]
Cells	ANCR	Interacts with EZH2 (RUNX2)	Regulates osteoblast proliferation and differentiation	[67]
Cells and animals	AK045490	Regulates Wnt/β-catenin pathway (RUNX2)	Regulates bone formation	[68]
Cells and Serum	MEG3	Regulates Wnt/β-catenin pathway	Regulates osteoblast proliferation and differentiation	[69]
Cells and Serum	H19	Sponging miR-19b-3p	Role in OP occurrence	[70]
Bioinformatic analysis	662 differentially expressed lncRNAs	Interact with different mRNAs	Regulates osteogenic differentiation	[71]

**Table 2:** Involvement of lncRNAs in osteoclastogenesis.

Sources	lncRNAs	Regulation Mechanism	Potential Role in Osteoclastogenesis	Study
EPC-derived exosomes	MALAT1	Sponging miR-124 (ITGB1)	Regulates osteoclast migration and differentiation	[72]
Cells	DANCR	Regulates Jagged1 and RANKL expression	Regulates osteoclast formation	[73]
Cells	NEAT1	Sponging miR-7 (PTK2)	Regulates osteoclast formation	[78]
Cells	TUG1	Regulates V-MafB	Regulates osteoclast differentiation	[79]
Cells and animals	MIRG	Sponging miR-1897 (NFATC1)	Regulates osteoclast formation	[80]
Cells	Bmncr	Downregulates osteoclast-related marker genes	Regulates osteoclast differentiation	[81]
Cells and animals	NRON	Regulates NFATC1 expression	Regulates osteoclastogenesis during orthodontic bone resorption	[82]
Bioinformatic analysis	RP11-498C9.17	Regulates genes mainly enriched in pathways regulating bone mineral density	Regulates osteoclastogenesis	[83]
Bioinformatic analysis	ENSG00000257764.2	Sponging miR-106a-5p (TIMP2)	Regulates osteoclast differentiation	[84]

**Table 3:** LncRNA GAS5 in OP and OS.

Disease	Sources	Pattern Expression Profile	Regulation Mechanism	Related Effects	Study
OS	Tissues and cells	↓	Sponging miR-23a-3p (PTEN/PI3K/AKT)	Regulates OS cell proliferation and invasion	[86]
OS	Cells	↓	Sponging miR-203a (TIMP2)	Regulates OS cell growth and metastasis	[87]
OS	Tissues and cells	↓	Sponging miR-221 (ARHI)	Regulates OS cell growth and EMT	[88]
OS	Tissues and cells	↓	Sponging miR-663a (RHOB)	Regulates OS progression	[89]
OS	Tissues and cells	↓	Sponging miR-663a (MYL9)	Regulates OS progression	[90]
OP	Tissues, cells, and animals	↓	Regulation of UPF1/SMAD7 axis	Regulates osteoblast differentiation	[91]
OP	Cells	↓	Sponging miR-498 (RUNX2)	Regulates osteogenic differentiation	[93]
OP	Serum	↑	/	/	[94]
OP	Plasma	↑	Downregulation of miR-21	Regulates apoptosis of osteoclasts	[95]

**Table 4:** LncRNA TUG1 in OS.

Disease	Sources	Pattern Expression Profile	Regulation Mechanism	Related Effects	Study
OS	Tissues, cells, and animals	↑	Sponging miR-153	Contributes to OS development	[104]
OS	Tissues, cells, and animals	↑	Sponging miR-143-5p (HIF-1α)	Regulates OS cell proliferation, metastasis, and angiogenesis	[105]
OS	Cells and animals	↑	Sponging miR-9-5p (POU2F1)	Facilitates OS tumorigenesis	[106]
OS	Tissues and cells	↑	Sponging miR-335-5p (ROCK1)	Regulates OS cell migration and invasion	[107]
OS	Tissues and cells	↑	Sponging miR-212-3p	Regulates OS cell proliferation and invasion	[108]
OS	Tissues and cells	↑	Sponging miR-132-3p (SOX4)	Regulates OS cell proliferation and apoptosis	[109]
OS	Tissues and cells	↑	Sponging miR-140-5p (PFN2)	Regulates OS cell proliferation, migration, and invasion	[110]
OS	Tissues and cells	↑	Sponging miR-144-3p (EZH2)	Regulates OS cell migration and EMT	[111]
OS	Cells and animals	↑	Regulation of MET/Akt signaling	Regulates cell growth and apoptosis under DDP treatment	[112]
OS	Cells	↑	Regulation of HK2	Regulates OS cell viability	[113]
OS	Cells	↑	Regulation of AKT signaling pathway	Regulates OS cell proliferation	[114]
OS	Tissues, cells, and plasma	↑	Regulation of RUNX2 expression	Regulates OS development	[115]

**Table 5:** LncRNA MALAT1 in OS, ES, and OP.

Disease	Sources	Pattern Expression Profile	Regulation Mechanism	Related Effects	Study
OS	Tissues and, cells	↑	Sponging miR-26a-5p (FOXO1)	Mediates OS cells proliferation and migration	[117]
OS	Tissues and cells	↑	Sponging miR-34a (CCND1)	Promotes OS progression	[118]
OS	Tissues, cells, and animals	↑	Sponging miR-202	Facilitates lung metastasis of OS	[119]
OS	Tissues, cells, and animals	↑	Sponging miR-129-5p (RET-Akt pathway)	Promotes OS metastasis	[120]
OS	Tissues, cells, and animals	↑	Sponging mir-144-3p (ROCK1/ROCK2)	Promotes OS cells metastasis and proliferation	[121]
OS	Tissues and cells	↑	Sponging miR-205 (SMAD4)	Promotes OS cells proliferation	[122]
OS	Tissues and cells	↑	Sponging miR-140-5p (HDAC4)	Regulates OS cells proliferation and apoptosis	[123]
OS	Tissues and cells	↑	Sponging miR-142-3p and miR-129-5p (HMGB1)	Promotes OS development	[124]
OS	Tissues and cells	↑	Sponging miR-376a (TGFA)	Promotes OS development	[125]
OS	Tissues and cells	↑	Sponging miR-129-5p (TGIF2)	Promotes proliferation, migration, and invasion of OS cells	[126]
OS	Tissues, cells, and animals	↑	Sponging miR-509 (Rac1/JNK Pathway)	Promotes the proliferation and metastasis of OS Cells	[127]
OS	Tissues and cells	↑	Regulating RhoA/ROCK pathway	Promotes OS development	[128]
OS	Tissues, cells, and animals	↑	Regulating E-cadherin and β-catenin expression	Supports OS metastasis	[129,130]
OS	Tissues and cell	↑	Regulating of PI3K/AKT signaling pathway	Regulates proliferation and metastasis of OS	[131]
OS	Cells and animals	↑	Regulating mTOR and HIF-1α	Induces pro-angiogenic effects in OS	[132]
ES	Cells and animals	↑	Regulated by SYK/cMYC	Contributes to the ES malignancy	[133]
ES	Cells and animals	↑	Regulated by TNC/Integrin α5β1/YAP axis	Regulates ES progression	[134]
OP	Bioinformatic studies	/	Regulating miR-22-3p-PTEN/ESR1/ERBB3/CSF1R/CDK6 and miR-32-3p-SP1/FZD6 axis	Possible role in OP biology	[135]
OP	Cells and animals	↓	Promoting the activation of the MAPK signaling pathway	Contributes to the OS onset and progression	[136]
OP	Cells and animals	↑	Sponging miR-34c (SATB2)	Mitigates the symptoms of OP	[137]

## Data Availability

Not applicable.
